# Biosocial and disease conditions are associated with good quality of life among older adults in rural eastern Nepal: Findings from a cross-sectional study

**DOI:** 10.1371/journal.pone.0242942

**Published:** 2020-11-30

**Authors:** Uday Narayan Yadav, Tarka Bahadur Thapa, Sabuj Kanti Mistry, Saruna Ghimire, Krishna Kumar Yadav, Godfred O. Boateng, Cathy O’Callaghan

**Affiliations:** 1 Centre for Primary Health Care and Equity, UNSW, Sydney, Australia; 2 School of Population Health, UNSW, Sydney, Australia; 3 Torrens University, Sydney, Australia; 4 Centre for Research, Policy and Implementation-Nepal, Biratnagar, Nepal; 5 James P Grant School of Public Health, BRAC University, Dhaka, Bangladesh; 6 Department of Sociology and Gerontology and Scripps Gerontology Center, Miami University, Oxford, OH, United States of America; 7 The University of Texas at Arlington, Arlington, Texas, United States of America; University of Zurich, SWITZERLAND

## Abstract

**Background:**

The ageing population in most low-and middle-income countries is accompanied by an increased risk of non-communicable diseases culminating in a poor quality of life (QOL). However, the factors accelerating this poor QOL have not been fully examined in Nepal. Therefore, this study examined the factors associated with the QOL of older adults residing in the rural setting of Nepal.

**Methods:**

Data from a previous cross-sectional study conducted among older adults between January and April 2018 in in rural Nepal was used in this study. The analytical sample included 794 older adults aged ≥60 years, selected by a multi-stage cluster sampling approach. QOL was measured using the Older People’s Quality of Life tool; dichotomized as poor and good QOL. Other measures used included age, gender, ethnicity, religion, marital status, physical activity, and chronic diseases such as osteoarthritis, cardiovascular disease, diabetes, chronic obstructive pulmonary disease (COPD), and depression. The factors associated with QOL were examined using mixed-effects logistic regression.

**Results:**

Seven in ten respondents (70.4%) reported a poor QOL. At the bivariate level, increasing age, unemployment, intake of alcohol, lack of physical activity as well as osteoarthritis, COPD and depression were significantly associated with a lower likelihood of a good QOL. The adjusted model showed that older age (AOR = 0.50, 95% CI: 0.28–0.90), the Christian religion (AOR = 0.38, 95% CI: 0.20–0.70), and of an Indigenous (AOR: 0.25; 95% CI: 0.14–0.47), Dalit (AOR: 0.23; 95% CI: 0.10–0.56), and Madheshi (AOR: 0.29; 95% CI: 0.14–0.60) ethnic background were associated with lower odds of good QOL. However, higher income of >NRs 10,000 (AOR = 3.34, 95% CI: 1.43–3.99), daily physical activity (AOR: 3.33; 95% CI: 2.55–4.34), and the absence of osteoarthritis (AOR: 1.9; 95% CI: 1.09–3.49) and depression (AOR: 3.34; 95% CI: 2.14–5.22) were associated with higher odds of good QOL.

**Conclusion:**

The findings of this study reinforce the need of improving QOL of older adults through implementing programs aimed at addressing the identified biosocial and disease conditions that catalyse poor QOL in this older population residing in rural parts of Nepal.

## Background

Globally, the population of older adults aged above 60 years has increased from 15% to 22% in 2015, the majority of which currently live in low- and middle-income countries (LMICs) [1]. This demographic transition is evident in Nepal [2, 3], a South Asian Country between India and China where in 2011, approximately 2.5 million of the Nepali population were 60 years and older [2]. The growth rate of the older population, at 3.5%, has exceeded the growth rate of the general population at 1.35% [4].

Global ageing has created a significant public health challenge for many countries, specifically LMICs, with scarce resources to address the social and health needs of its burgeoning population [1, 5]. Previous studies have linked poor quality of life (QOL) with age, level of education, economic status, ethnicity (particularly representing marginalised communities), religiosity, social support, physical activity, living arrangement and presence of co-morbidities [6–8]. Generally, older adults are at a higher risk of developing non-communicable diseases (NCDs), including cardiovascular diseases, diabetes, chronic kidney diseases, and mental disorders [9, 10]. This also has implications for the functional capacity of the individuals culminating in deleterious consequences for the general well-being and QOL of this population [11, 12]. Therefore, improving the QOL of older adults is an immense challenge in this 21^st^ century, where the proportion of older adults is increasing across the world.

According to the World Health Organization, QOL is the perceived position of an individual in their inhabitable value system and culture. It also relates to the goals, expectations, standards, and concerns of the individual’s life [13]. Moreover, QOL is the result of the effect of physical, functional, social, and emotional factors that lead to an individual’s wellbeing [14]. QOL has become an important outcome for many public health programs [15]. With an increase in the ageing population, maintaining the QOL of populations has become an important challenge [16].

As a signatory to the Sustainable Development Goals (SDG) for 2030, the government of Nepal has formulated a national policy on ageing and recently the “National Health Policy 2017” to address the needs of the Nepali older population [17]. However, the implementation of the policy is limited to a few programs such as the “Old–Age–Allowance,” “Jeshtha Nagarik Swashthopachar Kosh (Senior Citizens Health Facilities Fund)” and the free “Health Care Service Program” [18]. Many older adults, especially in rural areas, are deprived of such benefits simply because they are not aware that such programs exist, which may worsen their QOL relative to those benefiting from these programs. Previous studies on QOL among Nepali older adults have been limited to outpatient clinics [7] and nursing homes [19] in Nepal’s capital city of Kathmandu. This shows the dearth of evidence on QOL among the older adults in the community setting of south-eastern rural Nepal. To achieve the SDG, it is important to identify the key factors influencing the health and QOL of the older population and operationalise appropriate policy mechanisms to improve the wellbeing of older adults residing in rural Nepal. Therefore, this current study aimed to identify the state of QOL, and the factors associated with QOL among Nepali older adults in rural Nepal.

## Materials and methods

### Study designs and participants

Data from a previously conducted community-based cross-sectional study was used [20] in this study. The study was conducted among older adults, aged ≥60 years, living in the rural regions of *Morang* and *Sunsari* districts in Nepal. The data collection took place between January-April 2018. The sample size of 847 was calculated based on an unknown prevalence formula, i.e., Z^2^/4d^2^ [21]. Here, sampling error  =  5.0%, and Z = 1.96 at 95% CI. Further, a design effect of 2 and 5% non-response rate was added. A total of 794 eligible participants agreed to be interviewed in the study resulting in a response rate of 93.7%. Study participants were recruited from the community setting using a multi-stage cluster sampling approach, the details of which have been fully described in our previously published work [20].

#### Data collection and study variables

We used both semi-structured interviews and a validated survey questionnaire to collect data from the participants. The English version of the questionnaire was first translated to Nepali and then translated (forward-backward translation) back to English by two researchers to check the consistency of the instrument. Following this, 10 researchers and academics working in the area of public health were asked to verify the Nepali version of the tool in terms of readability and understandability of the tool contents. A pre-testing workshop was conducted in one rural municipality with 10 older adults and five health care workers to check the face-validity of the tool. The participants of the pre-testing workshop verified the tool with rephrasing of two sets of wording in the tool. In this study, Cronbach’s alpha for the quality of life scale was 0.75, which indicates acceptable reliability of the tool. The final Nepali version of the tool was administered for collection of the information.

### Outcome variable

QOL of the older adults was assessed by using the Older People’s Quality of Life (OPQOL) questionnaire [22], which is a novel instrument specifically designed to measure the QOL of older adults [23]. The OPQOL questionnaire has 35 questions that ask the participant to indicate the extent to which he/she agrees with each item in a Likert scale response (i.e., “strongly disagree”, “disagree”, “neither agree nor disagree”, “agree” and “strongly agree”). Each of the five possible answers is scored between one (“strongly disagree”) and five (“strongly agree”). The 35 items of this instrument consider the following aspects of QOL: life overall, health, social relationships and participation, independence, control over life and freedom, home and neighbourhood, psychological and emotional well-being, financial circumstances, leisure, activities, and religion. The cumulative score of the 35 items, which ranges from 35 to 175, provides the measure of overall QOL: a score of 70% and above is considered as a good QOL and scores less than 70% of are considered a poor QOL [24].

### Co-variates measurement

Independent variables included in the analysis were age; gender; ethnicity; religion; marital status; living arrangements; literacy status; occupation; monthly personal income; smoking habits; alcohol drinking habits; tobacco chewing habits; physical activity, depression and presence of any co-morbidities. These co-variate definitions and measurements are described in our previous work [25–27]. The 15-item Geriatric Depression Scale (GDS-15) was used to measure depression symptoms in this study [28]. The information on morbidities was collected from the medical records of the participants [26].

### Ethics

The study was approved [Reg.no.545/2017] by the Institutional Review Board of the Nepal Health Research Council, Government of Nepal, Ministry of Health, Kathmandu. Prior to the interviews, participants received an explanation about the study objectives and procedures, and the nature of voluntary participation. Written informed consent was then obtained from all literate participants, and thumb impressions were obtained from illiterate participants.

### Statistical analyses

We employed descriptive, bivariate and multivariable regression models for this study. First, descriptive analysis was carried out to present the distribution of background characteristics of participants, including their frequency, percentage, mean, standard deviation (SD) and range distributions. For the bivariate analysis, the chi-square (χ2) test was performed to compare the percentage of participants with varying QOL within different categories of variables at a 5% level of significance. Considering the nested nature of the survey data with possible variations among clusters (municipality), we performed a mixed-effect logistic regression model to assess the true association between the QOL and associated factors. Cluster variation was considered as a random effect and the rest of the variables were considered as fixed effects. The generalized estimating equation (GEE) was undertaken to estimate the parameters of the model while the exchangeable correlation structure within the clusters was employed. We retained in the final model only variables with a p-value of less than 0.25 in the bivariate model. Both unadjusted and adjusted odds ratios (AOR) with 95% confidence intervals (95% CI) are reported. All analyses were performed using Stata v. 13.0 (Stata Corp, College Station, TX).

## Results

### Descriptive statistics

A total of 794 older adults aged 60 years and above participated in the study. The mean age of the participants was 69.9 years; more than half (55.4%) were in their sixties (Table 1). The male to female ratio was close to unity (50.4% and 49.6%, respectively). A greater proportion of the participants were Hindu (78.7%) and illiterate (80.1%). Nearly 38% of the participants were of indigenous origin, and 34% were from the Madhesi or other ethnic groups. About half of the participants (53.8%) were married at the time of the survey. Regarding occupational status, 54.2% of the participants were not involved in any income-generating activities. As such, around half of the participants had a family income of 5000 Nepali Rupees (NRs) or less. More than three-quarters of the participants reported no physical activity at all (77.1%) and tobacco consumption history (76.8%), while only a quarter (25.1%) had alcohol drinking habits (Table 1). Depression (55.8%), osteoarthritis (41.7%), and chronic obstructive pulmonary disease (COPD) (15.4%) were the most prevalent health conditions among the participants (Table 2).

**Table 1:** Relationship between socio-demographic, lifestyle characteristics and quality of life.

				Quality of life				
		Total (N = 794)	%	Poor (N = 559)	%	Good (N = 235)	%	P value
Age (year, %)								
	60–69	440	(55.4)	293	(66.6)	147	(33.4)	0.009
	70–79	235	(29.6)	170	(72.3)	65	(27.7)	
	≥ 80	119	(14.9)	96	(80.7)	23	(19.3)	
Gender								
	Male	400	(50.4)	249	(62.3)	151	(37.8)	<0.001
	Female	394	(49.6)	310	(78.7)	84	(21.3)	
District								
	*Morang*	404	(50.9)	226	(55.9)	178	(44.1)	<0.001
	*Sunsari*	390	(49.1)	333	(85.4)	57	(14.6)	
Religion								
	Hinduism	625	(78.7)	437	(69.9)	188	(30.1)	<0.001
	Buddhism	19	(2.4)	4	(21.1)	15	(78.9)	
	Islam	125	(15.7)	104	(83.2)	21	(16.8)	
	Christianity	25	(3.2)	14	(56. 0)	11	(44.0)	
Ethnicity								
	*Brahmin/Chettri/Thakuri*	69	(8.7)	29	(42.0)	40	(57.9)	
	*Indigenous*	298	(37.5)	186	(62.4)	112	(37.6)	<0.001
	*Dalit*	157	(19.8)	119	(75.8)	38	(24.2)	
	*Madhesi and other ethnic groups*	270	(34.0)	225	(83.3)	45	(16.7)	
Marital status								
	Married	425	(53.5)	274	(64.5)	151	(35.5)	<0.001
	^1^Others	369	(46.5)	285	(77.2)	84	(22.8)	
Literacy								
	Illiterate	636	(80.1)	471	(74.1)	165	(25.9)	<0.001
	literate	158	(19.9)	88	(55.7)	70	(44.3)	
Past occupation								
	Employed	364	(45.8)	210	(57.7)	154	(42.3)	<0.001
	Unemployed	430	(54.2)	349	(81.2)	81	(18.8)	
Family monthly income								
	NRs < = 5000	381	(47.9)	312	(81.9)	69	(18.1)	<0.001
	NRs 5000 < = 10000	145	(18.3)	94	(64.8)	51	(35.2)	
	NRs > 10000	268	(33.8)	153	(57.1)	115	(42.9)	
Tobacco using habit								
	Never tobacco user	184	(23.2)	134	(72.8)	50	(27.2)	0.187
	Having tobacco use history	610	(76.8)	425	(69.7)	185	(30.3)	
Alcohol drinking habit								
	Never drinker	504	(63.5)	369	(73.2)	135	(26.8)	0.022
	Having alcohol drinking history	290	(36.5)	190	(65.5)	100	(34.5)	
Physical activity								
	No physical exercise at all	612	(77.0)	500	(81.7)	112	(18.3)	0.003
	Daily physical exercise	182	(22.9)	59	(32.4)	123	(67.6)	

^1^Others denotes widow/widower/divorced/separated/unmarried.

**Table 2:** Relationship between chronic disease condition and QOL.

				Quality of life				
		Total (N = 794)	%	Poor (N = 559)	%	Good (N = 235)	%	P value
Osteoarthritis								
	No	463	(58.3)	277	(59.8)	186	(40.2)	<0.001
	Yes	331	(41.7)	282	(85.2)	49	(14.8)	
CVD								
	No	775	(97.6)	542	(69.9)	233	(30.1)	0.065
	Yes	19	(2.4)	17	(89.5)	2	(10.5)	
Diabetes								
	No	752	(94.7)	529	(70.4)	223	(29.7)	0.881
	Yes	42	(5.3)	30	(71.4)	12	(28.6)	
COPD								
	No	672	(84.6)	455	(67.7)	217	(32.3)	<0.001
	Yes	122	(15.4)	104	(85.3)	18	(14.8)	
Depression								
	No	351	(44.2)	194	(55.3)	157	(44.7)	<0.001
	Yes	443	(55.8)	365	(82.4)	78	(17.6)	

Abbreviation: CVD- Cardiovascular disease, COPD- Chronic Obstructive Pulmonary Disease.

### Distribution of socioeconomic and lifestyle characteristic with QOL

Based on our classification, about two-thirds (70.4%) of our respondents reported poor QOL. The relationship between socio-demographic background, lifestyle characteristics and QOL are summarized in Table 1.

### Chronic condition and QOL

The Chi-square test for the relationship between chronic conditions and QOL showed that participants with prevalent osteoarthritis (p-value<0.001), COPD (p-value<0.001), and depression (p-value<0.001) had significantly poor QOL (Table 2).

### Summary of QOL indices

The numeric indices of QOL scores by different domains are presented in Table 3. The highest mean (SD) scores were in the religion/culture and independence domains with a score of 90.2 (16.4) and 72.7 (15.0), respectively. On the contrary, the health 46.2 (17.5) and psychological 46.3 (15.5) domains had the lowest mean scores among this population.

**Table 3:** Summary measures for different domains of quality of life.

Domains	Mean	SD	Min	Max
Life overall	61.6	17.4	20.0	100.0
Health	46.2	17.5	20.0	100.0
Social relationships	68.4	11.9	20.0	97.5
Independence	72.7	15.0	20.0	100.0
Home and neighbourhood	65.4	15.4	20.0	100.0
Psychological	46.3	15.5	20.0	100.0
Financial	69.4	13.6	20.0	100.0
Religion/culture	90.2	16.4	20.0	100.0
Overall	112.3	16.6	64.0	159.0

Abbreviation: SD- standard deviation.

### Association of risk factors with QOL

The multiple logistic regression model to assess the determinants of QOL is presented in Table 4. In the final model, age, religion, ethnicity, literacy, income, physical exercise, osteoarthritis, and depression were all factors significantly associated with good QOL. Individuals aged 70–79 years and ≥80 years old had 34% (AOR: 0.65; 95% CI: 0.49–0.88) and 50% (AOR: 0.50; 95% CI: 0.28–0.90) lower odds of having good QOL respectively compared to an individual aged 60–69 years. Compared to participants of the Hindu religion, higher odds of having good QOL were found in Buddhist participants (AOR: 3.08; 95% CI: 2.32–4.11); at the same time, lower odds were seen among Christian participants (AOR: 0.38; 95% CI: 0.20–0.70). Participants from Indigenous, Dalit and Madhesi/other ethnic groups had 75% (AOR: 0.25; 95% CI: 0.14–0.47), 77% (AOR: 0.23; 95% CI: 0.10–0.56) and 71% (AOR: 0.29; 95% CI: 0.14–0.60) lower odds of having good QOL respectively compared to participants from Brahmin/Chettri/Thakur ethnic groups.

**Table 4:** Multiple logistic regression model of the determinants of QOL.

	Variables	Crude OR			Adjusted OR	
		OR	95% CI	OR		95% CI
Age (year, %)						
	60–69	1.00		1.00		
	70–79	0.75	0.59–0.96	0.65		0.49–0.88
	> = 80	0.47	0.26–0.84	0.50		0.28–0.90
Gender						
	Male	1.00				
	Female	0.55	0.35–0.87	0.78		0.49–1.21
District						
	*Morang*	1.00		1.00		
	*Sunsari*	0.21	0.08–0.57	0.57		0.27–1.19
Religion						
	Hinduism	1.00		1.00		
	Buddhism	1.70	1.43–2.02	3.08		2.32–4.11
	Islam	0.75	0.48–1.18	0.97		0.57–1.65
	Christianity	0.59	0.34–0.99	0.38		0.20–0.70
Ethnicity						
	*Brahmin/Chettri/Thakuri*	1.00		1.00		
	*Indigenous*	0.28	0.13–0.59	0.25		0.14–0.47
	*Dalit*	0.22	0.10–0.50	0.23		0.10–0.56
	*Madheshi and other ethnic groups*	0.28	0.16–0.49	0.29		0.14–0.60
Marital status						
	Married	1.00		1.00		
	^1^Others	0.75	0.49–1.16	1.30		0.73–2.33
Literacy						
	Illiterate	1.00		1.00		
	literate	3.22	2.08–4.50	3.41		2.52–4.60
Occupation						
	Unemployed	1.00		1.00		
	Employed	0.58	0.41–0.82	0.71		0.42–1.19
^2^Income						
	NRs < = 5,000	1.00		1.00		
	NRs 5,000 < = 10,000	2.01	1.15–3.51	3.02		1.51–6.07
	NRs > 10,000	1.72	1.18–2.51	3.34		1.43–3.99
Tobacco using habit						
	Never tobacco user	1.00		Not taken in the model		
	Having tobacco use history	0.96	0.64–1.44			
Alcohol drinking habit						
	Never drinker	1.00		Not taken in the model		
	Having alcohol drinking history	1.06	0.72–1.58			
Physical activity						
	No physical exercise at all	1.00		1.00		
	Daily physical exercise	4.54	3.27–6.32	3.33		2.55–4.34
Osteoarthritis						
	Yes	1.00		1.00		
	No	2.17	1.20–3.93	1.95		1.09–3.49
CVD						
	Yes	1.00		1.00		
	No	2.54	0.75–8.54	2.68		0.67–10.72
Diabetes						
	Yes	1.00		Not taken in the model		
	No	1.15	0.51–2.60			
COPD						
	Yes	1.00		1.00		
	No	1.88	1.32–2.68	1.19		0.54–2.59
Depression						
	Yes	1.00		1.00		
	No	3.15	1.69–5.88	3.34		2.14–5.22

^1^Others denotes widow/widower/divorced/separated/unmarried.

^2^119 NRs approximates 1 US Dollar. Abbreviation: CVD- Cardiovascular disease, COPD- Chronic Obstructive Pulmonary Disease.

A literate older individual had almost three times higher odds of having a good QOL compared to an illiterate individual (AOR: 3.41; 95% CI: 2.52–4.60). The log-likelihood of good QOL also increased with increasing income. An older person with a family monthly earning of NRs 5,000 < = 10,000 or NRs > 10,000 had almost three times (AOR: 3.02; 95% CI: 1.51–6.07) or three and a half times (AOR: 3.34; 95% CI: 1.43–3.99) higher odds of having a good QOL compared to those who earned NRs < = 5,000. Good QOL was also associated with being physically active (AOR: 3.33; 95% CI: 2.55–4.34), having no osteoarthritis (AOR: 1.90; 95% CI: 1.09–3.49; compared to having osteoarthritis) and no depression (AOR: 3.34; 95% CI: 2.14–5.22; compared to having depression).

## Discussion

This study aimed to assess the QOL and its correlates among older adults in Eastern Nepal and found that seven in ten participants had poor QOL, which was significantly associated with age, socioeconomic status (SES), religion, ethnicity, physical activity, osteoarthritis, and depression.

The overall poor QOL observed in this study is consistent with previous studies from Nepal’s capital city of Kathmandu, where older patients in outpatient clinic [7] and nursing home [19] settings, had lower overall QOL scores. Previous studies, from international settings, are consistent with our findings [29, 30]. Further, a gradient decline in the odds of poor QOL was noted by increasing age group which is in line with a previous study from Nepal where age was inversely associated with QOL [31]. The declining QOL with age is plausible, given that older adults are at increased risk of chronic diseases and infection [9, 32]. Furthermore, age is associated with a progressive decline in muscle mass, strength, power, and physical performance [11, 12]. As a result, older adults have reduced mobility and functional capacity which ultimately influences overall wellbeing and lowers QOL in later life [11, 12].

A significant finding of this study is the role of SES and its implications for QOL. Better SES, as indicated by literacy and higher income, was associated with higher QOL among older adults in our study as well as others [33–35]. SES is considered one of the driving forces for existing health disparities globally [36]. Given the well-established relationship between SES and well-being in determining perceived health [37], mortality, and morbidity [38, 39]; the observed association with QOL was anticipated. Education increases health literacy and influences one’s ability to make informed decisions about their health and healthy behaviours [40]. Likewise, income increases purchasing capacity, access to health care, and affordability of everyday needs [41]. Together, education and income may determine one’s social status and the psychosocial advantages gained through social networks [41]. Specifically, a better SES may reflect the relative advantages, in terms of better economic and social positions, accumulated over the life course that may lead to better QOL in later life [42].

Similar to prior studies [43, 44], physical activity among the older adults was associated with QOL. The role of physical activity in the reduction of risk of chronic diseases and premature mortality, as well as the promotion of physical functionality and health in the general population, is well established [45–47]. However, within the confines of this population, this study provides evidence for the continual effect of physical activity over the life course. The pathways linking physical activity with QOL may be through the prevention of chronic diseases and the promotion of physical functioning and overall well-being. Previously, among older adults, several mediators such as better physical and mental health status, increased exercise, self-efficacy, increased physical self-worth, and reduced disability limitations, have been identified in the pathways between increased physical activity and QOL [43, 44].

In the context of Nepal, an individual’s ethnicity has a similar effect to their SES. Hence, it is not surprising to find that ethnicity was associated with QOL. Thus, compared to Brahmin/Chettri/Thakur ethnic groups, who are considered as part of the upper caste, participants from Indigenous, Dalit, and Madhesi/other ethnic groups had 75%, 77%, and 71% lower odds of having good QOL respectively. This is consistent with previous studies from Nepal, which have also suggested lower QOL scores among Dalits ethnic group compared to the upper castes, although the findings were statistically non-significant [7, 31]. Likewise, our finding aligns with the established notion that the Madhesi, Dalits, and Indigenous, being part of the marginalised groups, have poor outcomes in health and wellbeing, which is explained by their relative disadvantages in terms of low SES and lack of access to resources [48, 49]. Historically, these ethnic groups were considered disadvantaged in the society, in terms of their access to education and employment, and were discriminated against by the upper caste groups. Although, in recent years, such discrimination against them has been criminalised by the law and many commissions, such as the National Madheshi Commission, National Dalit Commission and National Indigenous Commission, have been established by the Government of Nepal to safeguard their rights, the quest for equality is still a long journey, especially in rural parts of Nepal where illiteracy is high and discriminatory practices from higher caste persons are long established [4]. These findings may suggest that the ethnic group you are born into may determine your QOL.

Another significant finding from this study is the significant association between religiosity and QOL. Here, compared to Hindu participants, QOL was higher among Buddhist participants and lower among Christian participants. Although the underlying explanations for the observed differences in QOL by religion are unknown, the literature does suggest that spirituality and religiosity are important components of QOL at any age [50]. Religious involvement may buffer stress and increase happiness, meaning, purpose and hope in life, which ultimately leads to better QOL [51]. Future studies have the opportunity to explain the observed association between religion and QOL; specifically, qualitative studies may be helpful to explore participants’ perceptions.

Physical and mental ailments were associated with lower QOL. The absence of osteoarthritis and depression was associated with a higher QOL score. Previously, low perceived QOL among patients with osteoarthritis was reported [52, 53]. The pain and limitations of daily living activities resulting from osteoarthritis may explain the observed reduced QOL [53, 54].

Our findings of an inverse association between QOL and depression are consistent with previous studies from Nepal and globally [30, 55, 56]. A meta-analysis of 24 studies reported moderate improvements in QOL following treatments for depression [57]. Depression may lower the QOL by impairing physical and social functioning, and overall health [58].

As with most studies, this study has some limitations. The participants were from eight rural municipalities of the Morang and Sunsari districts of Nepal; thus, the results can be only generalized to the settings. Secondly, social-desirability bias may have occurred as our findings relied on self-reported data. Further, the study adopted a cross-sectional design, which precludes any inferences of cause-effect relationships. The most important strength of this study includes a large sample size with more than a 90% response rate, strong methodology, and the adoption of Older People’s Quality of Life (OPQOL) questionnaire for the first time in Nepali settings.

## Conclusions

Our study found nearly three quarters of older adults residing in the community setting had poor QOL (particularly those in the very poor health and psychological domain) and we confirm that multiple factors are associated with it. Our findings emphasize the need for comprehensive community centered approaches aimed at improving the conditions that catalyse poor QOL in this study population.

## Supporting information

S1 Data (DTA)
